# Males and females with first episode psychosis present distinct profiles of social cognition and metacognition

**DOI:** 10.1007/s00406-022-01438-0

**Published:** 2022-07-08

**Authors:** M. Ferrer-Quintero, D. Fernández, R. López-Carrilero, I. Birulés, A. Barajas, E. Lorente-Rovira, A. Luengo, L. Díaz-Cutraro, M. Verdaguer, H. García-Mieres, A. Gutiérrez-Zotes, E. Grasa, E. Pousa, E. Huerta-Ramos, T. Pélaez, M. L. Barrigón, J. Gómez-Benito, F. González-Higueras, I. Ruiz-Delgado, J. Cid, S. Moritz, J. Sevilla-Llewellyn-Jones, Spanish Metacognition Group, A. Acevedo, J. Anglès, M. A. Argany, A. Barajas, M. L. Barrigón, M. Beltrán, I. Birulés, J. L. Bogas, A. Cabezas, N. Camprubí, M. Carbonero, E. Carrasco, R. Casañas, J. Cid, E. Conesa, I. Corripio, P. Cortes, J. M. Crosas, A. de Apraiz, M. Delgado, L. Domínguez, M. J. Escartí, A. Escudero, I. Esteban Pinos, C. Franco, C. García, V. Gil, R. Gonzalez-Casares, F. González Higueras, M. L. González-Montoro, E. González, E. Grasa, A.. Guasp, A. Gutierrez-Zotes, M. E. Huerta-Ramos, P. Huertas, A. Jiménez-Díaz, L. L. Lalucat, B. LLacer, R. López-Carrilero, E. Lorente, A. Luengo, N. Mantecón, L. Mas-Expósito, M. Montes, S. Moritz, E. Murgui, M. Nuñez, S. Ochoa, E. Palomer, E. Paniego, T. Peláez, V. Pérez, K. Planell, C. Planellas, P. Pleguezuelo-Garrote, E. Pousa, M. Rabella, M. Renovell, R. Rubio, I. Ruiz-Delgado, M. San Emeterio, E. Sánchez, J. Sanjuán, B. Sans, L. Schilling, H. Sió, M. Teixidó, P. Torres, M. A. Vila, R. Vila-Badia, F. Villegas, R. Villellas, S. Ochoa

**Affiliations:** 1 Parc Sanitari Sant Joan de Déu, Sant Boi de Llobregats, Dr. Pujades 42. Sant Boi de Llobregat 08830, Barcelona, Spain; 2 Departament de Psicologia Social I Psicologia Quantitativa, Facultat de Psicologia, Universitat de Barcelona, Barcelona, Spain; 3 Centro de Investigación Biomédica en Red de Salud Mental, Instituto de Salud Carlos III, Madrid, Spain; 4 Fundació Sant Joan de Déu, Esplugues de Llobregat Barcelona, Spain; 5 Departament de Psicologia, Facultat de Psicologia Clínica I de La Salut. Serra Hunter Fellow, Universitat Autònoma de Barcelona, Cerdanyola del Vallès, Bellaterra, Barcelona, Spain; 6 Department of Research, Centre d’Higiene Mental Les Corts, Barcelona, Spain; 7 Psychiatry Service, Hospital Clínico Universitario de Valencia, Valencia, Spain; 8 Hospital Universitari Institut Pere Mata, Institut d’Investigació Sanitària Pere Virgili-CERCA, Universitat Rovira i Virgili, Reus, Spain; 9 Department of Psychiatry, Hospital de la Santa Creu i Sant Pau, Institut d’Investigació Biomèdica-Sant Pau (IIB-Sant Pau), Barcelona, Spain; 10 Salut Mental Parc Taulí. Sabadell (Barcelona), Hospital Universitari–UAB Universitat Autònoma de Barcelona, Barcelona, Spain; 11 Neuropsiquiatria I Addicions, Hospital del Mar. IMIM (Hospital del Mar Medical Research Institute), Barcelona, Spain; 12 Departament of Psychiatry, University Hospital Virgen del Rocio, Seville, Spain; 13 Psychiatry Service, Area de Gestión Sanitaria Sur Granada, Motril, Granada, Spain; 14 Comunidad Terapéutica Jaén Servicio Andaluz de Salud, Jaén, Spain; 15 Unidad de Salud Mental Comunitaria Malaga Norte, Malaga, Spain; 16 Mental Health and Addiction Research Group. IdiBGi. Institut d’Assistencia Sanitària, Girona, Spain; 17 Department of Psychiatry and Psychotherapy, University Medical Center Hamburg, Hamburg, Germany; 18 Serra Húnter Fellow. Department of Statistics and Operations Research (DEIO), Universitat Politècnica de Catalunya BarcelonaTech (UPC), Barcelona, 08028 Spain; 19 Institute of Psychiatry and Mental Health, Health Research Institute (IdISSC), Clinico San Carlos Hospital, Madrid, Spain; 20 Institute of Mathematics of UPC - BarcelonaTech (IMTech), Universitat Politècnica de Catalunya, Barcelona, 08028 Spain; 21 Departament de Psicologia Clínica i de la Salut, Facultat de Psicologia, Universitat Autònoma de Barcelona, Bellaterra, Cerdanyola del Vallès, Spain; 22 COMSAL research group, FPCEE, Blanquerna Ramon Llull University, Barcelona, Spain; 23 GEIMAC, Institut de Neurociències, Universitat de Barcelona, Barcelona, Spain

**Keywords:** Sex differences, Profiles, Psychosis, Schizophrenia, Social cognition, Metacognition

## Abstract

Deficits in social cognition and metacognition impact the course of psychosis. Sex differences in social cognition and metacognition could explain heterogeneity in psychosis. 174 (58 females) patients with first-episode psychosis completed a clinical, neuropsychological, social cognitive, and metacognitive assessment. Subsequent latent profile analysis split by sex yielded two clusters common to both sexes (a Homogeneous group, 53% and 79.3%, and an Indecisive group, 18.3% and 8.6% of males and females, respectively), a specific male profile characterized by presenting jumping to conclusions (28.7%) and a specific female profile characterized by cognitive biases (12.1%). Males and females in the homogeneous profile seem to have a more benign course of illness. Males with jumping to conclusions had more clinical symptoms and more neuropsychological deficits. Females with cognitive biases were younger and had lower self-esteem. These results suggest that males and females may benefit from specific targeted treatment and highlights the need to consider sex when planning interventions.

**Supplementary Information:**

The online version contains supplementary material available at 10.1007/s00406-022-01438-0.

## Background

Sex differences in the onset and expression of psychosis are apparent since the first episode of psychosis (FEP) [1, 2]. Sex is one of the most predictive variables of clinical features in FEP [3] although this predictive power may be related to the large disparities that exist in other risk factors between the two sexes [4]. Men with psychosis have poorer premorbid adjustment, higher levels of substance abuse and dependence, and more negative symptoms [2, 4]. Furthermore, men usually exhibit worse social functioning [5] and male sex is a predictor of relapse after FEP [6]. Although the reasons behind better prognosis in females remain to be fully understood, there is cumulative evidence suggesting that disparities between both sexes start at a biological level, for instance at the genetic [7], neural [8] and hormonal [9] levels. Especially concerning the latter, a corpus of studies has shown the protective role of estrogens in psychosis [10] and its promise as a pharmacological treatment [11].

As well as biological variables, there are psychological constructs that deserve attention in their potential role for sex differences in psychosis, such as social cognition and metacognition. Patients with FEP experience significant deficits in social cognition [12] and metacognition [13]. Social cognition encompasses perception, interpretation, and information processing for adaptive social interactions [14], while metacognition refers to the spectrum of mental activities that involve reflection upon one's own, and others', thinking, and the synthesis of these phenomena into an integrated sense of self and others [15, 16]. Both social cognition and metacognition are important predictors of functional outcome when assessed globally [14, 17–19], but even specific subdomains of both constructs have distinct impacts on the disorder. The Jumping to Conclusions bias (JTC) has specific associations with neurocognition [20–23], inaccurate processing of social information [24], worse outcome [25], delusion forming and severity [21, 26, 27], and suicidal behavior [28]. Clinical insight has been related to treatment compliance, quality of life, depression, and symptoms among others [17, 29–31] but seems to be independent of neurocognition [32]. Attributional style has a clear influence in paranoia and persecutory delusions [33–35], and cognitive insight is related to depressive symptoms [36], and treatment compliance, symptoms, and quality of life [17].

Research exploring sex differences in social cognition and metacognition is inconclusive, probably due to the tendency to present averaged results [37]. A majority of studies have failed to find significant differences between sexes in social cognition [38–40] or metacognition [41, 42]. However, exploring differences in social cognition and metacognition beyond mean differences has often led to the discovery of important results. For instance, [18], looking for the effects of insight on symptoms found across symptom profiles, found a group characterized by positive symptoms and impaired insight that contained a majority of females [43]. Cobo et al. found that clinical insight correlated with different variables in each sex [42]. Similarly, García-Mieres et al. [43] found that females with psychosis present more extreme dichotomous thinking but a richer personal identity system than men [44]. Likewise, Salas-Sender et al. found that men and females with FEP responded differently to metacognitive training [45].

Differences in social cognition and metacognition in psychosis may not be apparent when comparing performance, but may be rooted in discrepancies in information processing. Data-driven methods permit capturing the heterogeneity in data in an exploratory manner. For example, Latent Profile Analysis (LPA) represents a promising technique to understand the possible configurations of social cognition and metacognition in males and females. LPA was designed to identify construct-based profiles [46], meaning that each profile captures latent attributes of a similar population. Furthermore, LPA is a person-based approach, which permits focusing on the characteristics of the individuals in predicting outcomes of interest [46].

In this work, we explored whether males and females with FEP present different profiles of social cognition and metacognition using LPA. As a second objective, we tested differences in demographic, clinical, and neuropsychological variables among the derived profiles. Given the exploratory nature of this study and the use of data-driven methods, we did not have a priori assumptions on the number of profiles and their characteristics or on the clinical differences among the profiles. We did, however, hypothesize that LPA is an adequate technique to detect configurations of social cognition and metacognition for each sex, and that profiles would have distinct clinical features.

## Methods

The design of the study and data collection stems from two research sources that had addressed the effectiveness of metacognitive training in people with FEP, under the register numbers NCT04429412 (conducted between 2015 and 2017) and NCT02340559 (conducted between 2012 and 2014). Data on the efficacy of metacognitive training of the clinical trial NCT02340559 have been published elsewhere [47]. Data of the clinical trial NCT04429412 have not been published yet. For the purposes of this work, only the baseline data of both clinical trials have been included in this study.

Participants from the two sources did not differ in age (*t*(170) = 0.91, *p* = 0.369, CI [ – 1.336, 3.578]), sex (χ^2^(1) = 0.749, *p* = 0.387) or diagnosis (χ^2^(5) = 3.671, *p* = 0.598).

### Participants

Participants were 174 (58 females) individuals with FEP. Patients were referred by clinicians at one of the community mental-health services of the following participant groups: Fundación Jiménez Díaz (Madrid), Servicio Andaluz de Jaén, Servicio Andaluz de Málaga, Centro de Salud Mental de Corporació Sanitària i Universitària Parc Taulí (Sabadell), Consultas externas del Hospital de Sant Pau (Barcelona), Centro de Higiene Mental Les Corts (Barcelona), Institut Pere Mata (Reus), Institut d´Assistència Sanitària Girona, Hospital Clínic de València and Parc Sanitari Sant Joan de Déu (PSSJD).

Inclusion criteria were as follows: (1) a diagnosis of schizophrenia, psychotic disorder not otherwise specified, delusional disorder, schizoaffective disorder, brief psychotic disorder, or schizophreniform disorder (according to DSM-IV-TR); (2) < 5 years from the onset of symptoms; (3) a score ≥ 4 in item delusions, grandiosity, or suspiciousness of PANSS in the last year; (4) age between 18 and 45 years.

Exclusion criteria were as follows: (1) traumatic brain injury, dementia, or intellectual disability (premorbid IQ ≤ 70); (2) substance dependence (3) Scores higher than 6 in the PANSS items “Hostility” or “Suspiciousness”.

### Measures

*Sociodemographic questionnaire:* Data on socio-demographic variables were collected on-site. Diagnosis and treatment were collected from the clinical history of the participants. We transformed the antipsychotic treatment to olanzapine defined daily dose (DDD) [48].

*Clinical measures:* The Positive and Negative Syndrome Scale (PANSS) [49, 50] was used to measure clinical and general symptoms. We used the 7-factor solution proposed by Emsley [51]. The Spanish version of the Scale Unawareness of Mental Disorders (SUMD) [52, 53] was used to measure unawareness of the mental disorder. Higher scores represent more unawareness of the mental disorder. We used the Rosenberg Self-Esteem Scale [54], where higher scores indicate better self-esteem.

*Metacognition:* The Beck Cognitive Insight Scale (BCIS) [55, 56] was used to measure cognitive insight. The BCIS is composed of the following two subscales: self-certainty and self-reflectivity, which are analyzed separately. Higher scores in self-reflectivity represent a higher ability to question one's beliefs. Higher scores in self-certainty represent more certainty in one's interpretations and misinterpretations. The Beads Task [57] was used to measure the JTC bias. Participants were shown a picture of two containers filled with 100 colored beads in reciprocal proportions. We used the following three trials with different conditions: a probabilistic trial with a 85/15 ratio, a second probabilistic trial with a 60/40 ratio, and a final trial with an affective condition in a 60/40 ratio. Participants were told that the computer had selected a container and that the goal of the task was to determine which container. To this end, participants were shown one bead at a time, and instructed to see as many beads as needed to guess which container the beads came from. Our outcome variable was the number of draws to decision in the three probabilistic conditions. Less than three draws to decision is considered indicative of presenting the JTC bias.

*Social Cognition:* The Internal, Personal, and Situational Attributions Questionnaire (IPSAQ) [58] was used to assess attributional style. We used the folloing two indexes: personalizing bias and externalizing bias. Personalizing bias refers to a tendency to blame others rather than circumstances for negative events. Externalizing bias refers to a tendency to attribute the causes of negative events to others or circumstances rather than to oneself [59]

The Faces Test [60, 61] was used to measure emotion recognition. A reduced version of The Hinting Task [62, 63] was used to measure theory of mind.

*Functional outcome:* The Global Assessment of Functioning (GAF) [64] was used to measure clinical and social functioning on a scale of 0–100. Higher scores represent better functioning.

*Neuropsychology:* The Wisconsin Sorting Card Test (WSCT) [65, 66] was used to assess flexibility and inhibition [67]. The Stroop Test (Stroop, 1935) was used to measure flexibility and inhibition. The Trail Making Tests (TMT-A and TMT-B) [68, 69] were used as a measure of visuomotor attention, sustained attention, speed, and cognitive flexibility. The Continuous Performance Test (CPT-II for Windows) [68, 69] was used to assess sustained attention and impulsivity. MATRICS CPT [70, 71] was used as a measure of attention in a subsample of the participants. We created the composite variable “Attention” by adding the D-prime scores of both measures standardized into T scores. All the neuropsychological variables are presented in T scores. The Weschler Adults Intelligence Scale (WAIS) [72] subtests Vocabulary and Digits were used to measure premorbid intelligence and verbal fluency, and working memory respectively. The scores are presented in their conversion to IQ.

### Statistical analysis

All descriptive analyses to explore the dataset were conducted using SPSS Version 22. We explored differences between sexes in all measures prior to conducting the Latent Profile Analysis using U-Mann Whitney tests. Effect size is reported using Cohen’s d.

Latent Profile Analysis (LPA) broken down by sex was carried out using R Version 3.5.3 [73], and in particular the R package *mclust* [74]. This method identifies profiles of individuals, called latent profiles, based on responses to a series of continuous variables. The number of latent profiles was determined by analyzing 2–6 group models in which the variables included were: Faces Test (total score), the Hinting Task (total score), the IPSAQ (personalizing bias and externalizing bias scores), the BCIS (self-reflectivity and self-certainty scores), and the three conditions of the Beads Task (trials to decision). Participants that lacked data in any of the aforementioned variables were excluded from the study. Of the initial 192 people that participated in the clinical trials, 174 were included in the LPA.Model selection to determine the optimal number of latent trajectories was performed according to the Bayesian Information Criterion (BIC) [75]. Additionally, we assessed variable importance by applying a classification tree via the R package *rpart* [76]. Model selection has been performed via Bayesian Information Criterion (BIC) for the specified LPA model numbers of clusters, which is fitted by EM algorithm [77] initialized by model-based hierarchical clustering [74, 78]. Additionally, the assessment of the variable importance was achieved building a CART model via recursive partitioning trees [79]. This ranking of variables is computed based on the corresponding reduction of predictive accuracy when the predictor of interest is removed using a measure of decrease of node impurity [80].

We used Kruskal–Wallis and Dwass-Steel-Critchlow-Fligner pairwise comparisons to calculate mean differences among the clusters. Effect size is reported using epsilon squared.

## Results

### Characteristics of the sample

A total of 174 patients with FEP were included in the analysis. Females were significantly older than males (*p* = 0.013) and had received significantly more education (*p* = 0.028). The samples differed in diagnosis (*p* = 0.004), depression as measured by the PANSS (*p* = 0.0016), theory of mind (*p* = 0.031), immediate recall (*p* = 0.009), short (*p* = 0.011) and long-term memory (*p* = 0.026), and the interference condition of the Stroop Test (0.05). We did not find any other significant differences between sexes. Full characteristics of the sample and comparisons by sex can be found in Supplementary Table 1.

### Profile analysis

#### Males

We identified three diagonal, variable volume, variable shape, coordinate axes orientation (VVI) profile profiles (i.e., diagonal profiles with variable shape, volume, and orientation aligned to the coordinate axes) according to BIC (BIC =  – 2854.815). Additionally, the CART classification tree assessed that the affective condition of the beads task (40%) and the 60–40 condition of the beads task (36%) were the most important variables in determining the profile structure.

The JTC profile (28.7%) included males that had one SD below the mean draws to decision in the three conditions of the Beads Task than the other two groups, suggesting a bigger tendency to present the jumping to conclusions bias.

The Indecisive profile (18.3%) presented a number of draws to decision of one SD above the mean in the three conditions of the Beads Task. The Homogeneous profile (53%) comprised participants who scored around the mean in all the variables examined.

**Fig. 1:**
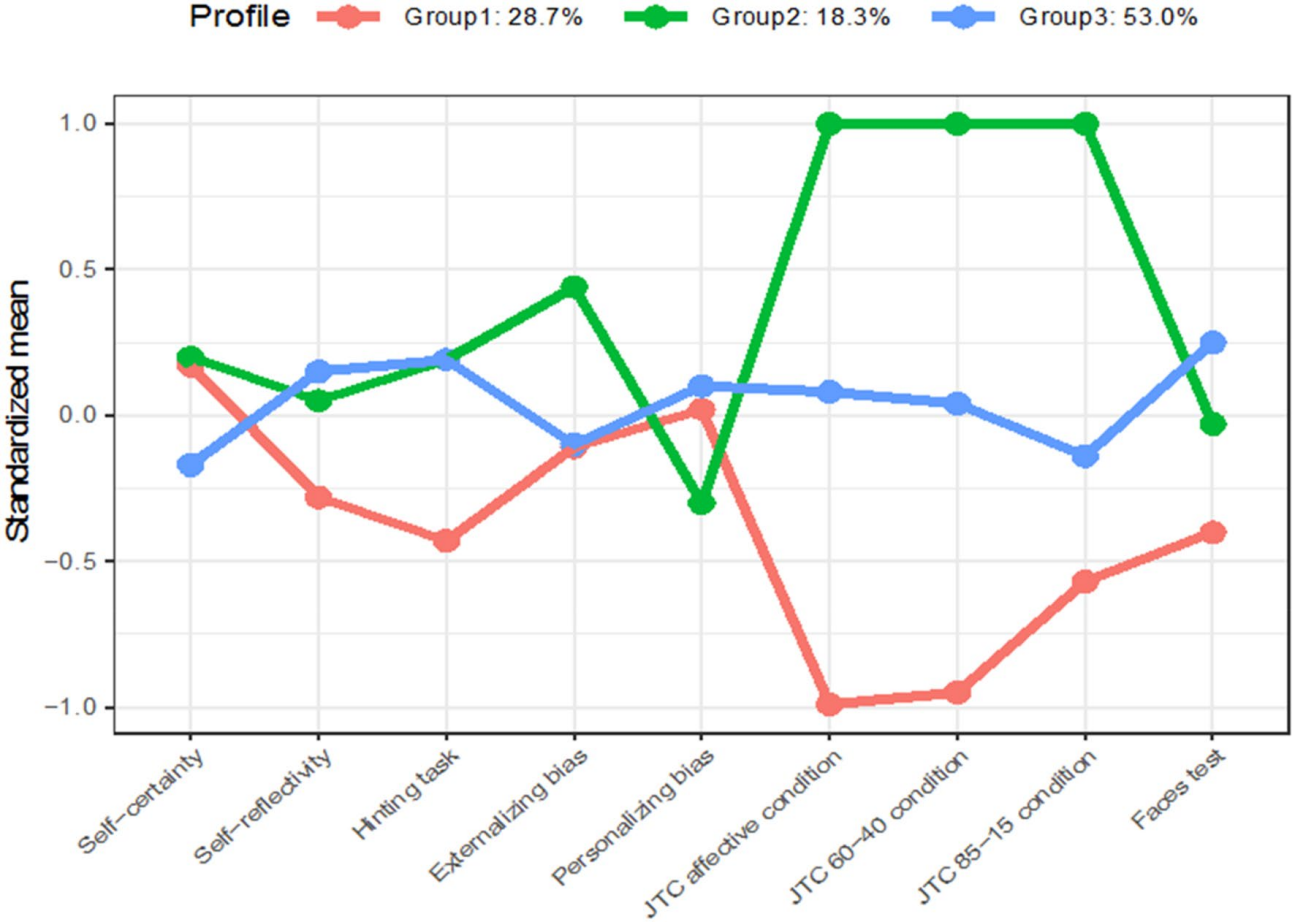
Profiles of each group in the male sample with standardized means in each of the variables included in the LPA. Group 1 refers to the JTC profile. Group 2 refers to the Indecisive profile. Group 3 refers to the Homogeneous profile

Figure 1 shows the graphic representation of each profile in the male group. Kruskal–Wallis tests yielded significant differences in positive (*p* = 0.03) and disorganized (*p* = 0.03) symptoms. Significant differences in positive symptoms did not survive subsequent pairwise comparisons. However, we found that males in the JTC profile had worse disorganized symptoms than males in the Homogeneous profile. Further, males in the JTC profile presented worse clinical insight than the other two profiles. We did not find other clinical differences.

As for neuropsychological variables, we found that males in the JTC profile scored worse than their counterparts in profiles Indecisive and Homogeneous in TMT-A and TMT-B, and worse than males in the Homogeneous profile in total errors of WSCT.

Males in the JTC profile scored better in our sustained attention measure than males in the Homogeneous profile. The mean scores of each variable included in the LPA and mean differences among profiles are presented in Table 1. Differences among the profiles in clinical and neuropsychological variables are displayed in Table 2.

**Table 1:** Mean scores of the social cognition and metacognition variables in each profile according to gender

	Males						Females					
	Profile 1	Profile 2	Profile 3				Profile 1	Profile 2	Profile 3			
	JTC	Indecisive	Homogeneous	*p*	Pairwise comparisons	ε^2^	Homogeneous	Indecisive	Cognitive bias	*p*	Pairwise comparisons	ε^2^a
	*N* = 33	*N* = 21	*N* = 61				*N* = 46	*N* = 5	*N* = 7			
	Mean (SD)	Mean (SD)	Mean (SD)				Mean (SD)	Mean (SD)	Mean (SD)			
Beads Task												
*85–15*	2.33 (1.16)	9.90 (6.14)	3.98 (1.70)	0.001	1 < 3, 1 < 2, 2 > 3	0.406	4.15 (2.53)	19.40 (0.89)	4.1 (3.18)	0.001	1 > 3, 2 > 3	0.241
*60–40*	3.12 (2.17)	15.14 (4.70)	8.18 (2.48)	0.001	1 < 2, 1 < 3, 2 > 3	0.647	6.72 (4.09)	18.00 (2.00)	6.71 (3.68)	0.002	1 > 3, 2 > 3	0.225
*Salient task*	2.88 (1.74)	13.52 (4.13)	7.72 (2.18)	0.001	1 < 2, 1 < 3, 2 > 3	0.685	6.96 (3.88)	18.20 (1.64)	6.71 (3.68)	0.002	1 > 3, 2 > 3	0.226
BCIS												
*Self-certainty*	9.12 (3.090)	9.12 (4.22)	7.78 (2.74)	0.083			8.07 (3.97)	8.40 (3.64)	8.00 (3.10)	0.964		
*Self-reflectivity*	14.33 (5.06)	16.0 (5.35)	16.48 (4.07)	0.101			14.26 (5.42)	15.20 (2.84)	20.29 (1.89)	0.009	1 < 3	0.165
Faces test	16.52 (2.81)	17.19 (1.91)	17.90 (1.50)	0.059			17.28 (1.73)	18.40 (0.54)	16.57 (1.39)	0.072		
Hinting task	1.33 (0.55)	1.61 (0.28)	1.62 (0.32)	0.053			1.70 (0.33)	1.60 (0.15)	1.52 (0.24)	0.104		
IPSAQ												
*Personalising bias*	1.25 (0.70)	1.04 (0.71)	1.30 (0.53)	0.205			0.092 (0.46)	1.30 (0.20)	2.75 (0.62)	0.0001	1 < 3	0.366
*Externalising bias*	0.45 (3.88)	2.52 (4.86)	0.23 (3.21)	0.099			2.54 (3.35)	1.00 (3.33)	– 4.86 (1.95)	0.0001	1 > 3, 2 > 3	0.3

**Table 2:** Mean scores and mean differences among the profiles in demographic, clinical and neuropsychological variables

	Males						Females					
	Profile 1	Profile 2	Profile 3				Profile 1	Profile 2	Profile 3			
	JTC	Indecisive	Homogeneous	*p*	Pairwise comparisons	ε^2^	Homogeneous	Indecisive	Cognitive bias	*p*	Pairwise comparisons	ε^2^
	*N *= 33	*N* = 21	*N* = 61				*N* = 46	*N* = 5	*N* = 7			
	Mean (SD)	Mean (SD)	Mean (SD)				Mean (SD)	Mean (SD)	Mean (SD)			
Age (years)	26.45 (6.70)	26.05 (8.06)	27.7 (6.87)	0.474			31.24 (7.86)	29.00 (5.97)	23.43 (7.85)	0.046	1 > 3	0.108
Education (years) (%)				0.001						0.001		
*Incomplete primary school*	18.2	14.3	5				6.5					
*Complete primary school*	24.2	28.6	11.7				10.9	20	28.6			
*Incomplete secondary school*	27.3	9.5	28.3				13.0	20	28.6			
*Complete secondary school*	18.2	23.8	33.3				23.9	20	28.6			
*Incomplete superior studies*	6.1	14.3	8.3				17.4	40	14.3			
*Complete superior studies*	6.1	9.5	13.3				28.3					
Antipsychotic dose (DDD)	14.17 (13.86)	9.03 (4.19)	18.73 (58.54)	0.372			22.38 (62.61)	9.58 (6.98)	12.51 (8.75)	0.703		
Diagnosis (%)				0.001						0.001		
*Schizophrenia*	48.48%	33.33%	52.46%				26.09%	60	28.57%			
*Psychotic disorder NOS*	12.12%	28.57%	36.07%				30.43%	20	14.29%			
*Schizoaffective disorder*	15.15%	4.76%	1.64%				10.87%	20	42.86%			
*Delusional disorder*	3.03%	14.29%	4.92%				13.04%		14.29%			
*Brief psychotic disorder*	12.12%	19.05%	3.28%				13.04%					
*Schizophreniform disorder*	6.03%		1.64%				6.52%					
Emsley factors												
*Positive symptoms*	17.97 (7.21)	17.71 (5.60)	14.97 (6.31)	0.021	1 > 3, 2 > 3	0.069	16.18 (6.42)	13.60 (3.91)	13.29 (4.72)	0.465		
*Negative symptoms*	16.18 (7.90)	16.76 (6.46)	15.46 (7.06)	0.680			14.36 (6.77)	15.80 (6.76)	15.29 (5.22)	0.986		
*Disorganised symptoms*	9.82 (4.28)	8.85 (3.62)	7.80 (3.32)	0.039	1 > 3	0.058	8.05 (3.85)	7.20 (3.27)	7.71 (2.36)	0.875		
*Excited symptoms*	6.15 (3.12)	5.52 (2.50)	5.41 (2.49)	0.408			5.43 (3.14)	4.20 (0.45)	4.43 (0.79)	0.472		
*Motor symptoms*	2.91 (1.87)	2.67 (1.28)	2.98 (1.44)	0.268			2.61 (1.11)	3.40 (2.19)	3.43 (1.27)	0.121		
*Depression*	4.52 (2.58)	4.76 (1.95)	4.08 (1.92)	0.333			5.09 (2.51)	6.40 (3.36)	5.29 (2.21)	0.603		
*Anxiety*	5.94 (2.38)	6.05 (2.27)	5.74 (2.28)	0.772			5.83 (2.42)	5.00 (1.22)	6.00 (3.42)	0.837		
GAF	60 (12.71)	57.10 (11.34)	60.11 (12.97)	0.538			60.00 (12.22)	54.2 (9.12)	60.43 (14.88)	0.467		
Rosenberg (total)	28.1 (6.83)	27.0 (5.20)	27.1 (6.12)	0.668			27.3 (5.42)	31.6 (8.02)	22.7 (6.52)	0.043	1 > 2, 2 > 3	0.110
BDI (total)	14.79 (9.35)	15.86 (7.61)	14.20 (9.43)	0.501			14.46 (9.12)	15.60 (12.12)	22.86 (7.49)	0.085		0.086
SUMD (global)	8.18 (3.86)	5.81 (3.63)	5.59 (3.02)	0.040	1 > 3	0.096	5.80 (3.97)	6.20 (5.07)	4.57 (2.15)	0.770		
WSCT (T)												
*Total errors*	39.71 (9.34)	46.90 (16.85)	47.46 (12.62)	0.024		0.072	44.98 (14.35)	43.60 (5.81)	41.29 (11.15)	0.968		
*Perseverative errors*	42.15 (8.10)	47.33 (17.21)	48.98 (12.58)	0.063			44.95 (15.13)	44.00 (8.43)	44.43 (7.44)	0.855		
*Non-perseverative errors*	40.25 (7.93)	45.33 (17.55)	46.61 (12.68)	0.063			45.45 (14.14)	43.40 (5.37)	39.71 (13.00)	0.704		
Stroop test (T)—interference	85.58 (19.11)	55.62 (11.76)	55.22 (12.21)	0.772			53.69 (10.71)	50.75(5.06)	51.29(14.77)	0.551		
WAIS-III (T)												
*Digits*	40.96 (7.96)	41.42 (9.67)	45.49 (9.93)	0.044	1 < 3	0.05	44.22 (9.26)	48.66 (6.41)	42.14 (10.11)	0.534		
*Vocabulary*	85.58 (19.11)	92.29 (24.92)	95.57 (18.32)	0,045	1 < 3	0.057	94.21 (21.07)	97.00 (7.58)	89.90 (27.45)	0.593		
Attention (T)	51.40 (12.11)	42.73 (14.20)	46.00 (6.12)	0.022	1 > 2	0.079	49.84 (13.19)	36.65 (15.60)	51.92 (11.76)			
TMT (seconds)												
*TMT-A*	73.19 (23.38)	64.24 (17.26)	62.31 (15.41)	0.049	1 > 3	0.055	65.94 (24.34)	66.25 (13.59)	64.58 (11.72)	0.664		
*TMT-B*	107.38 (81.88)	71.51 (18.91)	70.06 (23.28)	0.001	1 > 3	0.123	68.42 (20.13)	59.82 (14.00)	73.47 (17.11)	0.434		
Tavec												
*Immediate recall*	39.6 (9.20)	38.7 (9.22)	39.5 (9.92)	0.970			45.2 (12.4)	43.9 (15.1)	36.2 (7.20)	0.109		
*Short-term memory*	32.6 (12.3)	38.0 (16.8)	35.3 (17.7)	0.291			40.5 (13.3)	39.1 (9.58)	35.0 (13.2)	0.633		
*Long-term memory*	30.7 (14.2)	35.33 (17.68)	33.94 (16.26)	0.413			39.6 (14.2)	40.3 (10.5)	33.6 (18.0)	0.735		

#### Females

We identified three diagonal, variable volume, equal shape, coordinate axes orientation (VEI) profiles for females (i.e., diagonal profiles with variable volume, equal shape, and orientation aligned to the coordinate axes) according to BIC (BIC =  – 1443.49). The CART classification tree indicated that the most important variables in defining the profile structure were the Personalizing Bias (32%) and Externalizing Bias (23%) subscales of the IPSAQ.

The Homogeneous profile (79.3%) was the dominant group. Participants in this group scored around the mean in all the variables examined.

The Indecisive profile (8.6%) of the sample included participants with an excessive number of trials to decision in the Beads Task.

The Cognitive Biases profile (12.1%) was defined by high self-reflectivity, very low externalizing bias, and very high personalizing bias.

**Fig. 2:**
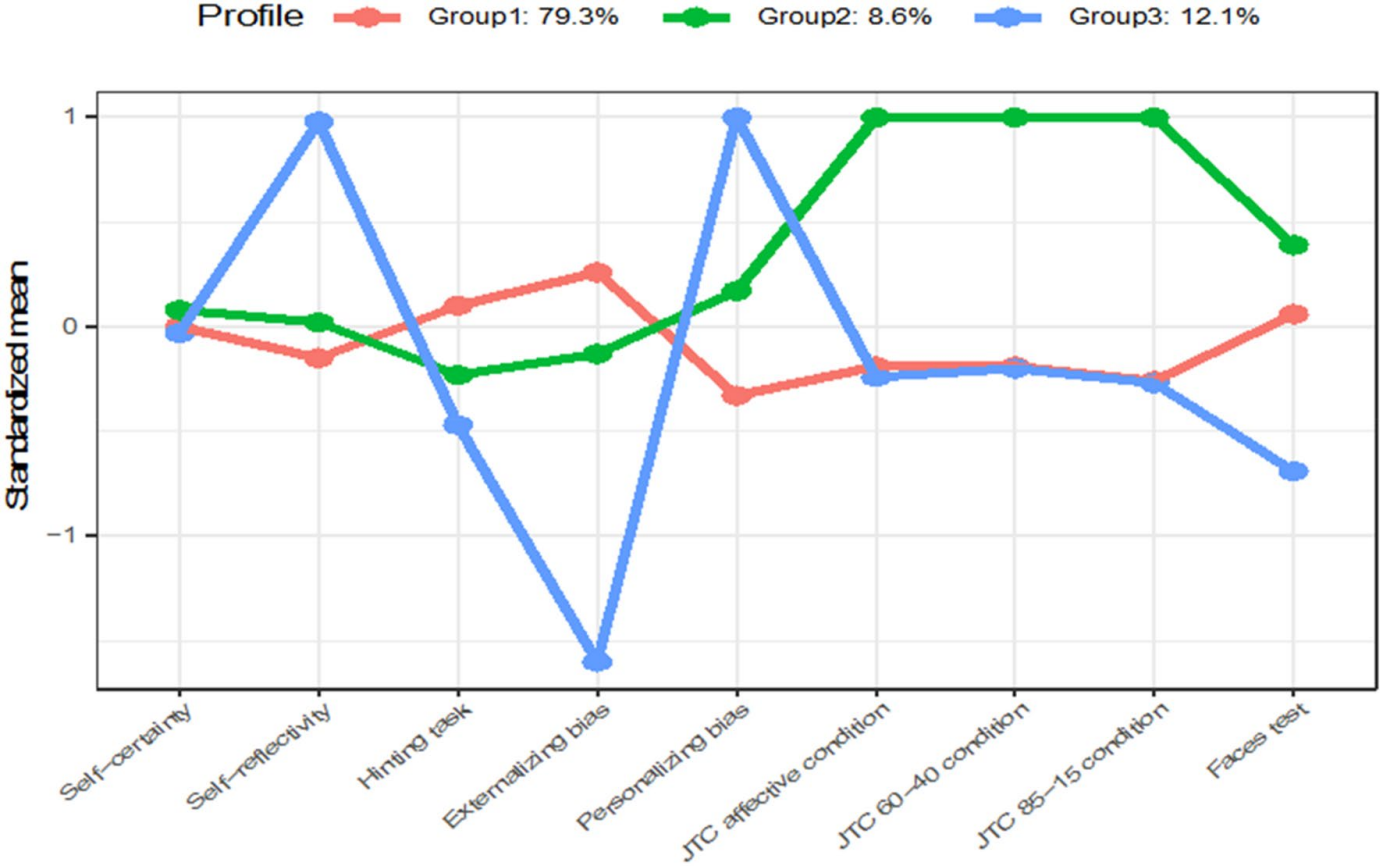
Profiles of each group in the female sample with standardized means in each of the variables included in the LPA. Group 1 refers to the Homogeneous profile. Group 2 refers to the Indecisive profile. Group 3 refers to the Cognitive Biases profile

Figure 2 shows the graphic representation of each profile in the female group. Kruskal–Wallis tests yielded significant age differences (*p* = 0.04) and self-esteem (*p* = 0.04). Subsequent pairwise comparisons indicated that females in the Homogeneous profile were significantly older than females in the Cognitive Bias profile.

The mean scores of each variable included in the LPA and mean differences among profiles are presented in Table 1. Differences among the profiles in clinical and neuropsychological variables are summarized in Table 2.

## Discussion

In this study, we conducted a latent profile analysis to obtain profiles of social cognition and metacognition in FEP according to sex. We identified three profiles in each sex. We found two profiles (Homogeneous and Indecisive) that were present in males and females, while we found two profiles (JTC and Cognitive Biases) that were specific to each sex. Consistent with our hypothesis, males and females with FEP present different profiles of social cognition and metacognition that are identifiable using LPA and that are associated with specific presentations of the disorder. Males in the Homogeneous profile seemed to have a more benign course of illness than males in the other profiles, particularly the JTC profile. Females in the homogeneous profile were older, had fewer depressive symptoms, and higher self-esteem than females in the Cognitive Bias profile.

These findings may have relevant clinical consequences, as our results suggest that having similar performance in all levels of social cognition and metacognition could be indicative of a more benign course of illness, although this explanation should be clarified in future research.

We found a second profile common to both sexes (Indecisive), characterized by average scores in most variables except for draws to decision, which were a standard deviation higher than the mean. Females in this profile only presented significantly better self-esteem than the other profiles. Males in this profile had more positive symptoms than males in the homogeneous profile but scored significantly better in attention than males in the JTC profile. This profile grouped the least proportion of participants both in males (18.3%) and females (8.6%). Participants in these groups seemed to have a clinical state similar to participants in the homogeneous profile. However, the importance of its traits cannot be neglected. Although to our knowledge the role of an excessive number of DTDs in the beads task has not been studied, one interpretation could be excessive metacognitive monitoring. Participants could be constantly evaluating whether they have enough information to make a decision, which could inhibit decision making [29]. The particularities of this profile indicate that subjects with this profile could benefit from a different therapeutic approach.

Males in the JTC profile had worse neuropsychological performance, more positive and disorganized symptoms, and worse clinical insight. These results are consistent with previous studies reporting the association between a higher tendency to present JTC and more positive symptoms [21] and worse neuropsychological deficits [20–22]. Some studies have suggested that JTC could likely be a consequence of pre-existing neuropsychological deficits [21, 23]. On the contrary, the association between clinical insight seems to be independent of neurocognitive abilities [32]. Nevertheless, the three constructs have been associated with poorer outcomes [17, 19, 25], indicating that males in this profile could have a more troubled course of the disease and worse functioning.

Females in the Cognitive Bias profile had more personalizing bias and self-reflectivity, but lower self-esteem than females in the other profiles. Further, we found a trend for significance in depression measured with BDI. Females in the Cognitive Bias profile scored higher in depression than the other two profiles. This presentation seems consistent with the insight paradox [30], a phenomenon in which more self-reflectivity is positively associated with more depression and lower self-esteem [36].

Depression, self-esteem, and personalizing bias have been found not only to be closely associated with persecutory ideation and paranoia [33, 34, 58], but also with the severity of paranoia in subjects with FEP [35]. Females in this profile have more self-reflectivity, indicating that they have a better ability to reflect upon their processes. This ability may lead to a better awareness of their symptoms and difficulties, which could decrease self-esteem and increase depression. Ultimately, to preserve their self-esteem, females in this profile could blame other persons for negative events, which may, in turn, increase paranoid symptoms and perpetuate symptoms. This explanation, however, remains speculative as this study did not explore causality.

We note that females in the Homogeneous profile were older than those in the Cognitive Bias profile. Although examining hormonal differences between the profiles is beyond the aim of this work, it is possible that differences in estrogen levels are partially responsible for the clinical presentation of each profile. This hypothesis should be examined in future research.

Our work has several limitations.

First, our sample was not balanced in sex, which could have hampered our statistical power. Likewise, the sample size of each profile varied greatly. Therefore, although we used non-parametric tests to determine mean differences, some significant differences may not have been detected. Similarly, we did not conduct post-hoc analysis, as the comparisons presented in this work are qualitative comparisons based on the graphical representation of the clusters. We did not have a control group. Therefore, whether these profiles appear in the general population, the extent of the impairment, and cut-off scores could not be calculated. We used a cross-sectional design that did not allow testing profile stability. There are other possible predictors of profile membership that were not collected in the present work, such as differences in personality[81], that should be considered in future studies. These limitations notwithstanding, this is the first work yielding evidence of sex profiles in social cognition and metacognition. Future research confirming our profile solution, profile membership predictors, and illness course according to profile and sex are recommended, as well as understanding therapeutic components of interventions that are more adequate to specific sexes and profile presentations.

There are relevant clinical implications to our work. A first implication is that males that present JTC and females that present higher self-reflectivity in conjunction with personalizing bias may have a worse presentation of the disorder. Importantly, the JTC and other cognitive biases are modifiable [82]. Therefore, the early identification of cognitive and metacognitive profiles may help clinicians deliver early targeted treatment, what could have a beneficial effect in prognosis.

Patients with different profiles of social cognition and metacognition may respond differently to therapeutic approaches. A study assessing sex differences in response to metacognitive treatment in a sample with FEP [45] reported that females improved more in cognitive insight, personalizing bias, and general symptoms than males. Conversely, males improved more in the salient condition of the Beads Task, but not females. Our results are consistent with them in that our profiles follow the same direction as their findings and further support them in that future studies should study which contents of metacognitive interventions could be more beneficial according to sex and profile of impairment.

While all the profiles could benefit from therapies that target metacognition, males could benefit from boosting sessions aimed at correcting the JTC, while females could benefit from boosting sessions directed to modify cognitive insight and attributional biases. Moreover, males that present JTC may find optimal treatment in combining neurocognitive training with metacognitive therapy. Predictors of profile membership and possible illness trajectories emerge in our work as promising topics for future research. Longitudinal studies assessing the prognosis of each profile and profile stability are encouraged to help confirm these exploratory findings.

## Supplementary Information

Below is the link to the electronic supplementary material.

Supplementary file1 (XLSX 14 KB)
